# Phosphodiesterase 7 Inhibition Preserves Dopaminergic Neurons in Cellular and Rodent Models of Parkinson Disease

**DOI:** 10.1371/journal.pone.0017240

**Published:** 2011-02-24

**Authors:** Jose A. Morales-Garcia, Miriam Redondo, Sandra Alonso-Gil, Carmen Gil, Concepción Perez, Ana Martinez, Angel Santos, Ana Perez-Castillo

**Affiliations:** 1 Instituto de Investigaciones Biomédicas, Consejo Superior de Investigaciones Científicas CSIC-UAM, Arturo Duperier, 4 and Centro de Investigación Biomédica en Red sobre Enfermedades Neurodegenerativas (CIBERNED), Madrid, Spain; 2 Instituto de Química Médica, CSIC, Juan de la Cierva, Madrid, Spain; 3 Departamento de Bioquímica y Biología Molecular, Facultad de Medicina, Universidad Complutense de Madrid, Madrid, Spain; Centre national de la recherche scientifique, University of Bordeaux, France

## Abstract

**Background:**

Phosphodiesterase 7 plays a major role in down-regulation of protein kinase A activity by hydrolyzing cAMP in many cell types. This cyclic nucleotide plays a key role in signal transduction in a wide variety of cellular responses. In the brain, cAMP has been implicated in learning, memory processes and other brain functions.

**Methodology/Principal Findings:**

Here we show a novel function of phosphodiesterase 7 inhibition on nigrostriatal dopaminergic neuronal death. We found that S14, a heterocyclic small molecule inhibitor of phosphodiesterase 7, conferred significant neuronal protection against different insults both in the human dopaminergic cell line SH-SY5Y and in primary rat mesencephalic cultures. S14 treatment also reduced microglial activation, protected dopaminergic neurons and improved motor function in the lipopolysaccharide rat model of Parkinson disease. Finally, S14 neuroprotective effects were reversed by blocking the cAMP signaling pathways that operate through cAMP-dependent protein kinase A.

**Conclusions/Significance:**

Our findings demonstrate that phosphodiesterase 7 inhibition can protect dopaminergic neurons against different insults, and they provide support for the therapeutic potential of phosphodiesterase 7 inhibitors in the treatment of neurodegenerative disorders, particularly Parkinson disease.

## Introduction

Parkinson disease (PD) is one of the most common progressive neurodegenerative disorder, affecting around 1% of the elderly population. Typical symptoms of this disease are muscle rigidity, bradykinesia, resting tremor and postural instability. At the cellular level, PD is characterized by the loss of dopamine-containing neurons in the substantia nigra pars compacta (SNpc) although neuropathology can extend into other brain regions [1]. The cell death leads to the loss of dopamine in areas where these neurons project, causing the described symptoms. The main known risk factor is age, however susceptibility genes including α-synuclein, leucine rich repeat kinase 2 (LRRK-2), and glucocerebrosidase (GBA) have shown that genetic predisposition is another important causal factor in a 10% of diagnosed patients. There is currently no cure and no effective disease-modifying therapy. The dopamine replacement therapy in clinical use is only palliative; leading to temporarily limited improvement of clinical symptoms, and the chronic treatment with dopaminergic drugs have severe side effects as bradykinesia. Consequently, new approaches to treat Parkinson disease are needed to find disease's modifying agents that may delay or stop the neuronal death.

Neuroinflammation has been increasingly recognized as a primary mechanism involved in PD pathogenesis [2], [3]. Loss of dopamine-producing neurons in PD is accompanied by inflammation in surrounding support glial cells. Activation of microglia has been demonstrated in SN and striatum from postmortem PD brains and in PD animal models [4], [5], [6]. This inflammatory state in glial cells leads to the production of toxic substances, including cytokines such as IL-1β, IL-6, and TNF-α, that further damage neurons, leading to a cycle of inflammatory damage that ultimately worsens the progression of the disease. New evidence in experimental animals indicates that blocking the signaling pathways in glial cells responsible for turning on neurotoxic genes dramatically decreases damage to dopaminergic neurons. Unfortunately, current therapies do not address this neuroinflammation problem, being focused on ameliorating the symptoms of dopamine loss rather than on the underlying causes of injury to dopaminergic neurons. Targeting the signaling pathways in glial cells responsible for neuroinflammation represents a promising new therapeutic approach designed to preserve remaining neurons in PD patients, thereby extending the window of efficacy of existing symptomatic drugs in order to better maintain quality of life. Given the evidence for neuroinflammation in PD, agents with anti-inflammatory effects have been investigated for their neuroprotective potential [7].

Different studies have suggested that cyclic AMP (cAMP) levels might play an important role in neuroprotection and in the neuroinflammatory response [8], [9] thus control of the levels of this nucleotide could trigger the regulation of the pathological neuroinflammatory process and, consequently, to delay the progression of neurodegenerative disorders, such as PD. Intracellular cAMP levels depend on one hand on their synthesis by adenylyl cyclases and, on the other hand, on its degradation by cyclic nucleotide 3′, 5′-phosphodiesterases (PDEs) [10], [11]. Hence, PDEs have recently emerged as important drug targets for regulating several diseases [12].

The PDEs comprise a family of 21 members, which have been so far classified into 11 groups, according to their sequence homology, cellular distribution, and sensitivity to different PDE inhibitors [11], [12], being some of them expressed on central nervous system [13]. PDE7 is a cAMP-specific PDE, which is insensitive to a PDE4 inhibitor, Rolipram [10], [11] and it has been recently demonstrated that can be a target for the control of neuroinflammation [14]. The PDE7 family is composed of two genes, PDE7A and PDE7B. High mRNA concentrations of both PDE7A and PDE7B are expressed in rat brain and in numerous peripheral tissues, although the distribution of these enzymes at the protein levels has not been reported. Within the brain PDE7A mRNA is abundant in the olfactory bulb, hippocampus, and several brain-stem nuclei [15]. The highest concentrations of PDE7B transcripts in the brain are found in the cerebellum, dentate gyrus of the hippocampus and striatum [16], [17]. There is very little information regarding the physiological functions regulated by PDE7. It has been shown that PDE7 is involved in pro-inflammatory processes and is necessary for the induction of T-cell proliferation [18]. In addition, specific inhibitors of PDE7 have been recently reported as potential new drugs for the treatment of brain diseases [19]. However, a detailed analysis of the effect of these compounds on normal central nervous system function as well as in pathological conditions have yet to be described.

Several years ago, our research group was the first one in reporting the first PDE7 selective inhibitors [20]. Since then, a lot of efforts have been done to increase potency and selectivity of this kind of compounds, conforming a great variety of diverse chemical compounds with interesting pharmacological profiles [21]. We have recently reported a new and diverse chemical family of PDE7 inhibitors, the quinazolines ones, discovered by using a ligand-based virtual screening [22]. Moreover, the biological profile of these new thioxoquinazolines showed that they are useful compounds to decrease the inflammatory activation in a T-cell line [23].

**Figure 1:**
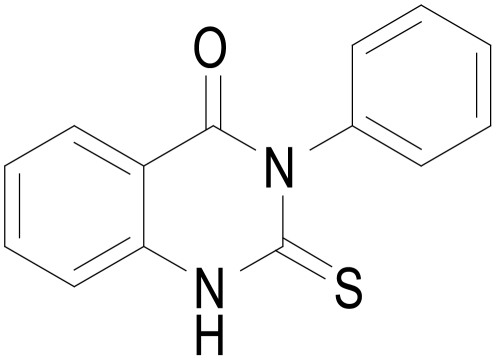
Structure of the PDE7 inhibitor used in the experiments, the quinazoline derivative S14.

In the present study, we demonstrate for the first time, that PDE7 inhibition enhances neuroprotection and diminishes neuroinflammation in well-characterized cellular and animal models of PD. In addition, treatment of adult rats with the blood brain barrier permeable PDE7 inhibitor named S14 (Phenyl-2-thioxo-(1H)-quinazolin-4-one, Figure 1) significantly protects dopaminergic neurodegeneration and improves motor function in LPS-lesioned animals. Lastly, we also show that its effects are mediated by the cAMP/PKA signaling pathway. As such, these findings identify PDE7 as a potential therapeutic target for the treatment of Parkinson Disease.

## Results

### Expression of PDE7

**Figure 2:**
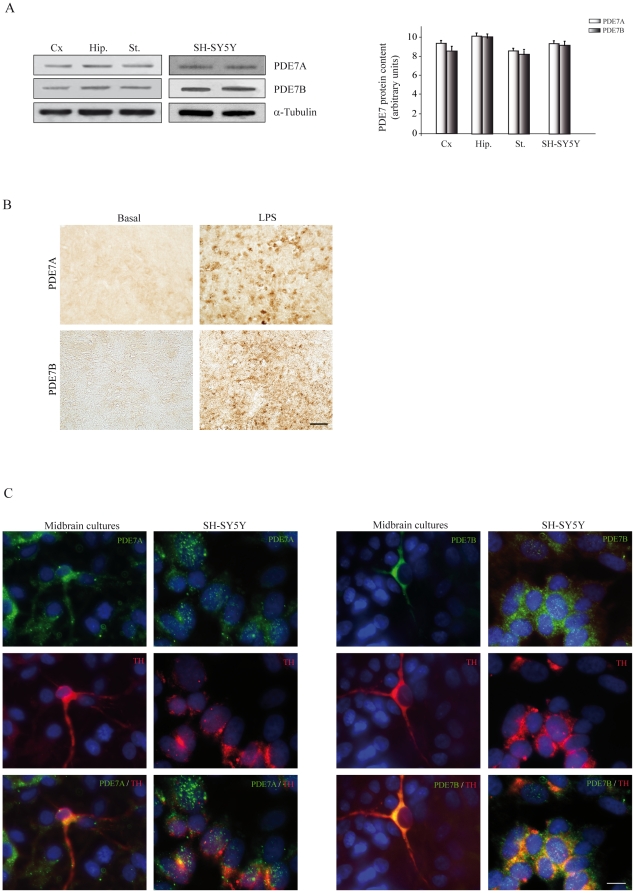
Western blot and immunocytochemical analysis of PDE7A and PDE7B. (**A**) Representative Western blot and quantification analysis showing expression levels of PDE7A and PDE7B in different brain regions and in the dopaminergic cell line SH-SY5Y. Cx, cerebral cortex; Hip, hippocampus; St, striatum. (**B**) Inmunohistochemical analysis of PDE7A and PDE7B expression in the *substantia nigra pars compacta* (SNpc) of adult rats. Figure also shows the expression of both isoenzymes 72 h after lipopolysaccharide (LPS, 10 µg) injection in this area. Scale bar, 25 µm. (**C**) Immunofluorescence analysis of PDE7A and PDE7B expression (green) and tyrosine hydroxylase (TH, red) in the dopaminergic cell line SH-SY5Y and in primary mesencephalic cultures. Representative results of at least three independent experiments are shown. Scale bar, 10 µm. Nuclei were counterstained with DAPI (blue).

We first analyzed whether PDE7 was expressed throughout the central nervous system of the adult rat. As can be seen in Figure 2A, significant levels of PDE7A and PDE7B were detected in different brain regions including the striatum. In the case of the SNpc, we found, by immunohistochemistry studies, that the levels of PDE7A and PDE7B are low in the basal state. However, they were notably increased after LPS injection (Figure 2B). These results are of interest since these genes have been related to inflammation [18]. Moreover, the increased observed after LPS injury support our data showing an important role for PDE7 inhibitors as neuroprotective agents of dopaminergic neurons. Additionally, both isoforms of PDE7 are expressed in the SH-SY5Y neuroblastoma cell line and in primary rat mesencephalic cultures (Fig. 2C). Besides, double immunocytochemistry studies clearly show that TH positive cells expressed PDE7A and PDE7B.

### PDE7 inhibition protects neuronal SH-SY5Y cells from 6-hydroxydopamine (6-OHDA)-induced death

**Figure 3:**
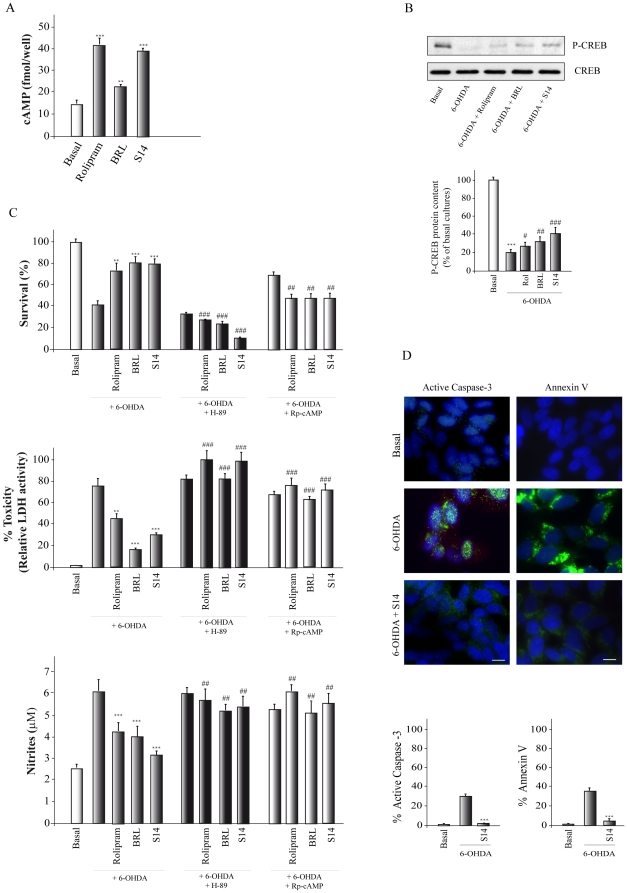
Effect of S14 on 6-OHDA-induced SH-SY5Y cell death. Cells were treated with Rolipram (30 µM), BRL50481 (BRL, 30 µM), or S14 (10 µM) as indicated in Methods. (**A**) Intracellular levels of cAMP in cells treated during 1 hr with the indicated compounds. ^**^p<0.01; ^***^p<0.001 *versus* non-treated (basal) cells. (**B**) Representative Western blot showing phosphorylation of CREB after incubation of cells with 6-OHDA (35 µM) for 16 h in the presence or absence of the indicated compounds. A specific anti-phospho-CREB antibody was used. The use of an antiserum that does not discriminate between CREB and phospho-CREB (bottom panel) indicates that the total levels of CREB are not affected by the treatments. Quantification analysis are shown. ^***^p<0.001 *versus* non-treated (basal) cells; ^#^p<0.05, ^##^p<0.01, ^###^p<0.001 *versus* 6-OHDA-treated cells. Rol, Rolipram (**C**) Cell viability, cytotoxicity and nitrite production were measured as indicated in Methods. Some cultures were pretreated with the protein kinase A inhibitor H89 or the cAMP antagonist Rp-cAMP. Values represent the mean ± SD of six replications in three different experiments. ^**^p<0.01; ^***^p<0.001, *versus* 6-OHDA-treated cells; ^##^p<0.01, ^###^p<0.001 *versus* the values obtained in the absence of H89 or Rp-cAMP (**D**) Apoptotic levels were determined by active caspase 3 (green) and Annexin V-FITC (green) immunodetection. Representative images of at least three independent experiments are shown. Scale bar, 10 µm. Nuclei were counterstained with DAPI (blue). Quantification of active caspase 3 and Annexin V-FITC-positive cells is shown. ^***^p<0.001 *versus* 6-OHDA-treated cells.

The human dopaminergic neuronal cell line SH-SY5Y possesses many qualities of substantia nigra neurons [24] and is therefore widely used as a model to study the death of dopaminergic neurons. Since S14 has been described as a PDE7 inhibitor, we first analyzed whether this compound could increase cAMP levels on SH-SY5Y cells. To this end, cells were treated for 1 h with S14 and two well-known PDE4 and PDE7 inhibitors, Rolipram and BRL50481, respectively, and cAMP levels were analyzed by ELISA. Figure 3A shows that, as expected, Rolipram and BRL50481, were able to elevate the levels of cAMP in these cultures. Treatment with S14 also resulted in a significant increase in the levels of cAMP. We next analyzed the phosphorylation state of the *cAMP response element-binding protein* (CREB), a known target of the cAMP/PKA signaling pathway. As shown in Figure 3B, treatment of SH-SY5Y with Rolipram, BRL50481 or S14, together with 6-OHDA, resulted in an increase of phosphorylated CREB levels.

We then examined the effect of S14 on the cell death induced by 6-OHDA exposure. As shown in Figure 3C, 6-OHDA treatment resulted in a loss of viability, as assessed by a decline in (3-(4,5-dimethylthiazol-2-yl)-2,5-diphenyl tetrazolium bromide) (MTT) and a significant elevation in lactate dehydrogenase (LDH) level (Fig. 3C), as compared with control untreated cells. Incubation with the PDE7 inhibitor quinazoline compound S14 afforded significant protection against 6-OHDA-induced cell death lowering elevated LDH levels by as much as 50% and reversing the decline in MTT by 22%. This neuroprotective effect was mimicked by BRL50481 and by Rolipram. S14 has an IC50 of 5.5 µM on PDE7A, five times more potent than its inhibition on PDE4D (IC50 = 22 µM) [22]. Quinazoline derivative S14 does not inhibit PDE3 (3% of inhibition at 10 µM) therefore preventing the compound from possible cardio toxic effects. Hence, the results obtained here suggest that S14 protects the human dopaminergic neuronal cell line SH-SY5Y from cell death through an inhibition of the PDE7 enzyme.

Toxicity induced by 6-OHDA was also accompanied by an increase in nitrite production (Fig. 3C, lower panel), and its concentration was brought toward normality after S14 treatment, indicating that this drug blocks 6-OHDA-induced oxidative stress, which leads to free radical generation.

### Activation of PKA by cAMP is required for S14-induced neuroprotection of SH-SY5Y

The most common intracellular target of cAMP is PKA. PKA activation is responsible for many of the actions attributed to cAMP [25]. Nonetheless there are other effects of this nucleotide, which are not mediated by PKA [26], [27]. We therefore investigated if PKA activation is required for the neuroprotective actions of S14. To this end, SH-SY5Y exposed to 6-OHDA and pretreated or not with Rolipram, BRL50481 or S14, were treated with the PKA inhibitor H89 or the specific membrane-permeable inhibitor of PKA activation adenosine 3′,5′-cyclic monophosphorothioate Rp-isomer (Rp-cAMP). As shown in Figure 3C, both compounds prevented the increase in cell viability and the decrease in nitrite liberation elicited by the three PDE inhibitors, suggesting that the cAMP/PKA pathway mediates their effects on SH-SY5Y cells.

Lastly, apoptosis was determined by measuring the levels of active caspase 3 and Annexin V analysis (Fig. 3D). Our results indicate that 27% of the SH-SY5Y cell population was positive for caspase 3 staining within 16 h after treatment with 6-OHDA and that this effect was almost completely reversed by the treatment with the S14 compound. Annexin V-FITC analysis also showed a significant decrease in the number of apoptotic cells in those cultures treated with S14 (Fig. 3D). These results suggest that S14 is rescuing SH-SY5Y cells from 6-OHDA-induced apoptosis.

### PDE7 inhibition protects cultured primary mesencephalic cells from lipopolysaccharide- and 6-OHDA-induced cell death

**Figure 4:**
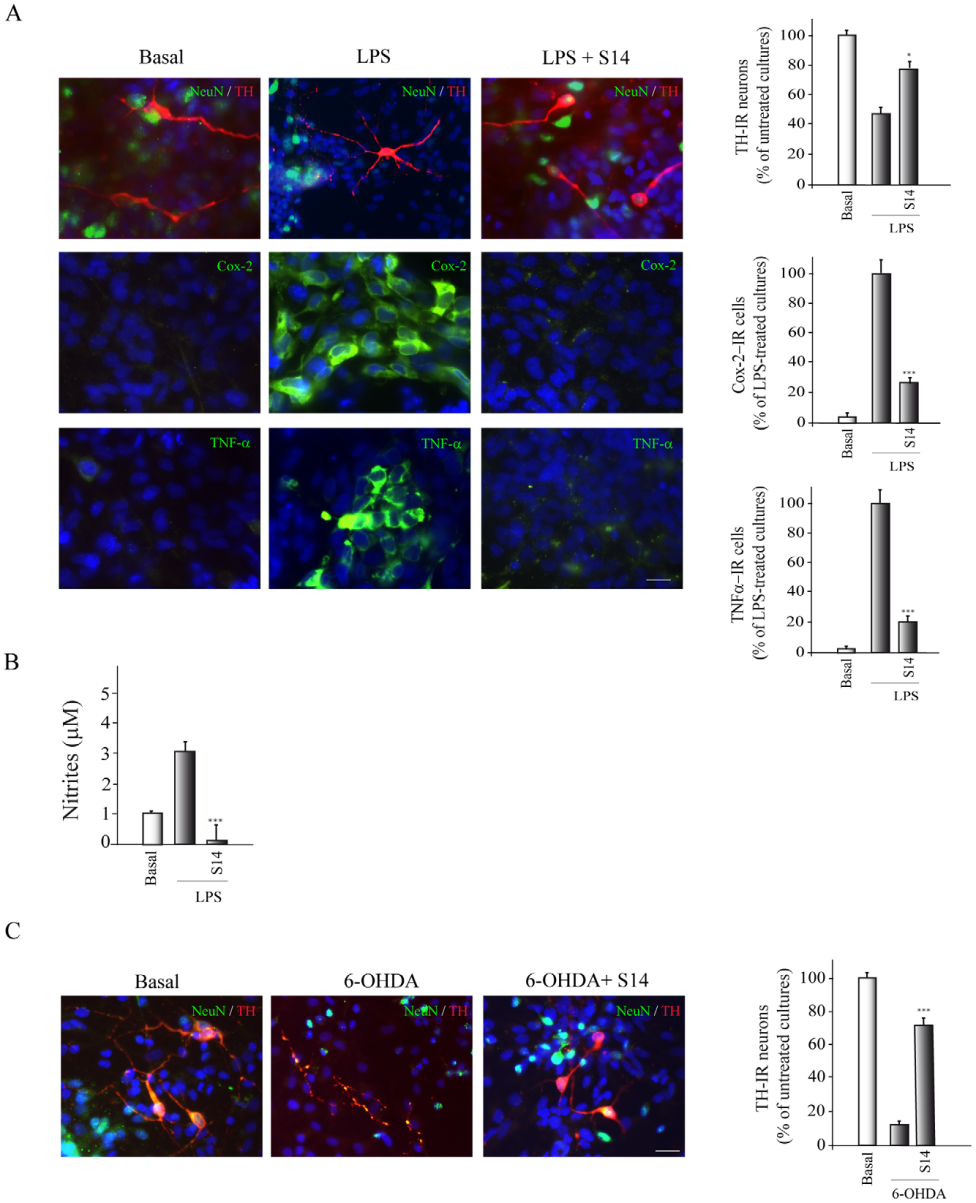
Effect of S14 on cell death and inflammation processes in mesencephalic cell cultures induced by incubation with LPS or 6-OHDA. (**A**) Rat primary mesencephalic cultures were treated with LPS (1 µg/ml) in the absence or presence of S14 (10 µM) and the expression of TH, COX-2 and TNF-α was evaluated by immunofluorescence analysis using specific antibodies, as described in Methods. Representative results of three independent experiments are shown. Scale bars, 20 µm. Nuclei were counterstained with DAPI. Quantification of the numbers of immunoreactive cells was performed as described in Methods. Values represent the mean from three different experiments and twenty independent fields (≥50 cells/field) per culture. ^*^p<0.05; ^***^p<0.001 *versus* LPS-treated cells. (**B**) Nitrite production was evaluated by the Griess reaction. Values represent the mean ± SD of six replications in three different experiments. ^***^p<0.001, *versus* LPS-treated cells (**C**) Rat primary mesencephalic cultures were treated with 6-OHDA in the absence or presence of S14 and the expression of TH^+^ cells was evaluated by immunofluorescence analysis using specific antibodies, as described in Methods. Representative results of three independent experiments are shown. Scale bars, 20 µm. Nuclei were counterstained with DAPI. Quantification of the numbers of immunoreactive cells was performed as described in Methods. Values represent the mean ± SD from three different experiments and twenty independent fields (≥50 cells/field) per culture. ^***^p<0.001 versus 6-OHDA-treated cells.

We next examined whether PDE7 inhibition could also have neuroprotective effects on primary ventral mesencephalic cultures. These cultures are known to be vulnerable to LPS treatment, resulting in a loss of neuronal viability [28]. The viability of mesencephalic cell cultures, known to be rich in dopaminergic neurons, was determined by quantifying tyrosine hydroxylase (TH) immunoreactivity after exposure to LPS. Treatment with this endotoxin decreased the number of TH^+^ cells by 42% (Fig. 4A). S14 addition significantly preserved TH^+^ cells from LPS toxicity. No significant difference in the number of DAPI-positive nuclei was found among the treated cultures (data not shown). We also analyzed whether S14 affected the LPS-induced expression of TNF-α and COX-2, two well known proinflammatory agents. As shown in Figure 4A, incubation of primary mesencephalic cultures with S14 completely abrogated the induction of TNF-α and COX-2 expression after LPS treatment, suggesting that the protection observed by S14 could be exerted, at least in part, through an effect upon inflammatory reaction of microglial cells present in the cultures. These results were further corroborated by measuring nitrite liberation to the culture medium (Fig. 4B). LPS treatment resulted in an increase in the concentration of nitrites in the cultures medium, which was significantly prevented by S14. In fact the levels of nitrites in the S14-treated cultures were even lower that those detected in control non-treated cells.

The neuroprotective effects of S14 were also tested after exposure to the dopaminergic toxin 6-OHDA. As expected, 6-OHDA significantly decreased TH-positive cells (80%) (Fig. 4C). Addition of S14 to the cultures conferred a robust protection against 6-OHDA-mediated cell loss.

### Neuroprotective role of PDE7 inhibition in an *in vivo* model of PD

**Figure 5:**
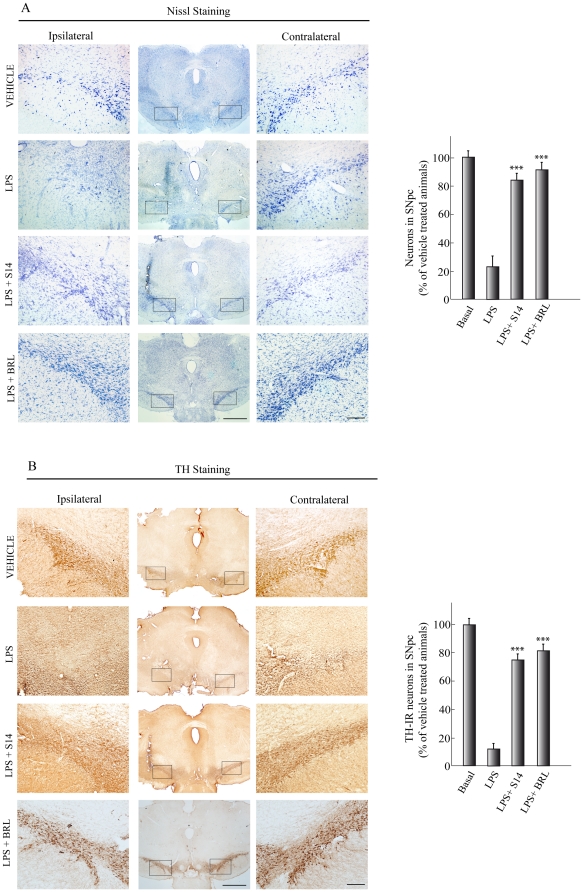
Effect of S14 on dopaminergic cell death *in vivo*. LPS (10 µg) or vehicle was injected unilaterally into the adult *substantia nigra pars compacta* (SNpc) of adult rats. A group of animals also received S14 (20 nmol) or BRL50481 (BRL, 60 nmol) together with LPS. After 72 h the brains were removed and tissue sections were processed for (**A**) Nissl staining to label neurons or (**B**) tyrosine hydroxylase (TH) immunoreactivity to label dopaminergic neurons. Scale bars, 500 µm. Insets scale bars, 100 µm. Quantification of the numbers of neurons in A or TH-immunoreactive (IR) cells in B is shown. Values represent the mean ± SD, expressed as a percentage of vehicle-treated animals, from three different experiments, four animals/experiment/experimental group, and five independent sections per animal. ^***^p<0.001 versus LPS-treated animals.

Given the in vitro anti-inflammatory and neuroprotective effects described above, we then assessed the efficacy of S14 in a well-characterized rodent model of PD. LPS injection into the SNpc of rodents induces dopaminergic cell loss and microglial activation [29], [30]. To this end, adult rats were injected unilaterally in the SNpc with vehicle, LPS, or LPS plus S14 and were killed 72 h after injection. Histological analysis were used to evaluate the extend of dopaminergic cell loss and microglial activation in the SNpc of the different groups of animals. A significant preservation of dopaminergic neurons was found in S14-injected rats compared with abundant dopaminergic neuron damage after injection with LPS (Fig. 5). Quantitative studies showed a decrease of 85%, compared with the vehicle-injected rats, in the number of dopaminergic neurons in the SNpc after LPS injection. In contrast, in the S14-treated group, only a moderate decrease (25%) in dopaminergic cell number was observed 3 days after LPS injection. These results extend the observations made in vitro and suggest that treatment of LPS-injected animals with S14 results in an almost complete prevention of dopaminergic injury. In addition, we also analyzed the effect of BRL50481, a well characterized PDE7 inhibitor, on this same model of PD. Our results showed that BRL50481 has similar neuroprotective and anti-inflammatory effects as S14. Only a 20% decrease in the number of dopaminergic neurons (Figure 5B) was observed in rats injected with this compound, compared to 85% found in LPS-treated animals.

**Figure 6:**
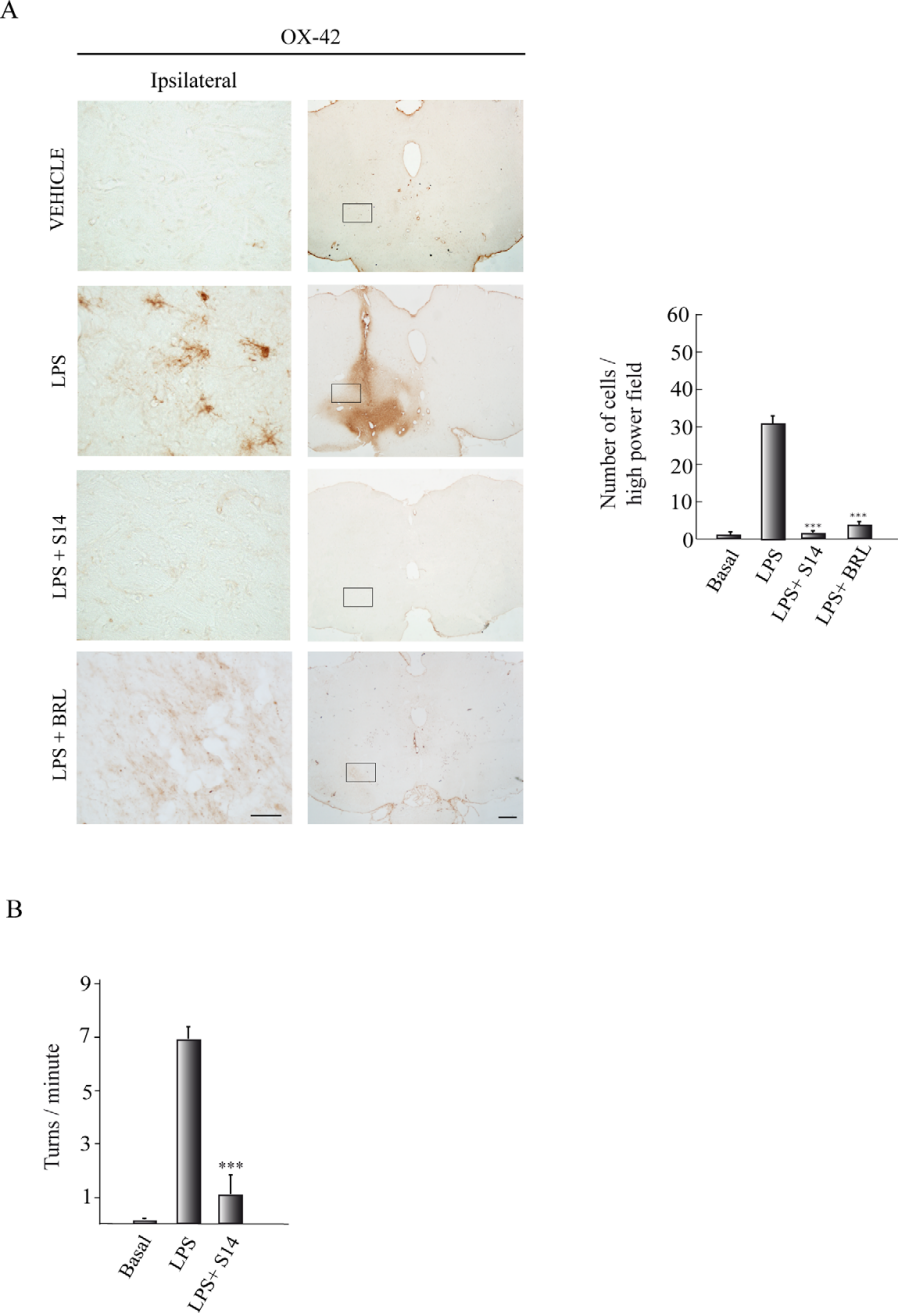
Effect of S14 on *in vivo* inflammation and rotational behavior. Lipopolysaccharide (LPS, 10 µg) or vehicle was injected unilaterally into the adult *substantia nigra pars compacta* (SNpc) of adult rats. A group of animals also received S14 (20 nmol) or BRL50481 (BRL, 60 nmol) together with LPS. (**A**) After 72 h the brains were removed and tissue sections were processed for CD11b (OX-42) immunoreactivity lo label activated microglia. Scale bars, 500 µm. Insets scale bars, 100 µm. Quantification of the reactive cells is expressed as the mean ± SD, from three different experiments, four animals/experiment/experimental group, and five independent sections per animal. ^***^p<0.001 versus LPS-treated animals. (**B**) Behavioral analysis. Three weeks after treatment apomorphine-induced rotations were analyzed in control, LPS-injected, and LPS+S14-injected rats. Values represent the means ± SD from three different experiments. ^***^p<0.001, *versus* LPS-injected animals.

One of the events that take place in the SNpc after LPS injury is the activation of microglial cells, which is in part responsible for the dopaminergic cell degeneration. Microglial cells (identified as OX-42-positive cells) were very scarce in the contralateral part of LPS-injected animals and in the SNpc of vehicle-injected animals (Figure 6A). Seventy-two hours after LPS injection, a high OX-42 immunoreactive signal was clearly observed in the SNpc. This strong microgliosis was completely absent in the animals treated with the quinazoline PDE7 inhibitor S14. Also, BRL50481 treatment of LPS-injured rats completely abrogated the microgliosis observed in the LPS-treated group (Figure 6A). Altogether, these results reinforce our hypothesis that PDE7 could be an important target for neuroprotection of dopaminergic neurons.

Finally we analyzed the effects of S14 treatment on rotational behavior by assessing the behavioral changes in hemi-parkinsonian rats. To this end, three weeks following LPS lesion rats were injected with apomorphine, which is known to induce contralateral rotational behavior in denervated animals. Figure 6B shows that the LPS-treated rats exhibited 7 contralateral turns per minute following an administration of apomorphine. Rats lesioned with LPS and treated with S14 showed a significant improvement (only 1 turn per minute) after apomorphine. Vehicle-treated animals showed no contralateral rotational behavior. Immunohistochemistry analysis also showed that, three weeks after LPS administration, dopaminergic cell death was significantly attenuated in the group treated with S14 (data not shown).

## Discussion

In this study, we have demonstrated, for the first time, that inhibition of PDE7 induces neuroprotection of human dopaminergic neuronal cells SH-SY5Y and of primary mesencephalic cultures and attenuates the production of nitrites and proinflammatory agents. Our data also show that inhibition of PDE7 results in an inhibition of microglial activation and has neuroprotective effects on the nigrostriatal system in an *in vivo* model of PD. In addition, the neuroprotective effect of PDE7 inhibition appears to be mediated by the cAMP/PKA signaling pathway. These results suggest that inhibition of PDE7 can represent a new therapeutic approach for the treatment of PD and other neurodegenerative disorders in which inflammation processes are involved. Thus, PDE7 inhibitors may represent a new generation of valuable drugs.

We initially analyzed the neuroprotective and anti-inflammatory effects of the PDE7 inhibitor S14 in the human dopaminergic cell line SH-SY5Y and in primary mesencephalic cultures. Human neuroblastoma cells exposed to 6-OHDA are used as *in vitro* model for PD, due to similar cellular processes that occur in the degenerating dopaminergic neurons [31]. We show that S14 significantly attenuates 6-OHDA-induced neuronal cell death and nitrite liberation in the SH-SY5Y neuronal cell line and in mesencephalic cultures. These effects are accompanied by an elevation of intracellular cAMP levels, indicating that also in dopaminergic neurons the activity of PDE7 is important in governing cellular cAMP concentration. The mechanism of action of this compound seems to be the inhibition of the PDE7 enzyme, the subsequent activation of the cAMP/PKA signaling pathway and the activation of the transcription factor cAMP response element-binding protein (CREB) by phosphorylation. It is known that cAMP can activate at least three different signaling pathways within cells. The first one to be characterized and the most extensively studied rely on the activation of PKA, which then phosphorylates different substrates including transcription factors such as CREB. However, cAMP can also stimulate the guanine nucleotide exchange protein Epac, which in turn activates the GTPase Rap-1 [32]. Other pathway identified as activated by cAMP includes another guanine nucleotide exchange protein called CNrasGEF, which directly activates Ras [33]. Yet, our results showing a reversion of the anti-inflammatory and neuroprotective effects of S14 by both Rp-cAMP and H89 (a specific inhibitor of PKA activation), support the notion that S14 specifically activates cAMP-dependent PKA activation.

These neuroprotective actions of PDE7 inhibition are in accordance with previous findings showing that cAMP signaling pathway might inhibit cell death in various neurodegenerative disorders. Previous work has demonstrated a clear involvement of PKA in neuroprotection [34], [35]. Absence of CREB in developing brain results in generalized cell death, whereas postnatal disruption of this transcription factor triggers progressive neurodegeneration [36]. Also, it has been shown that CREB is necessary for neuronal survival and axonal growth in different neuronal populations [9]. Of note, inhibition of cAMP signaling pathway has been suggested to contribute to Hungtinton disease pathology [37], [38], [39]. Our results add new and important data establishing that elevation of intracellular cAMP levels through inhibition of PDE7 promotes protection of dopaminergic cells and has potent anti-inflammatory effects.

To evaluate the translational relevance of the aforementioned cellular effects, the anti-inflammatory and neuroprotective actions of direct administration of S14 into the brain were assessed in a classical rodent model of PD. Research in the last years has unveiled an important role for neuroinflammation in the degeneration of the nigrostriatal dopaminergic pathway that constitutes the pathological basis of PD. Neuroinflammation is characterized by the activation of glial cells that release various cytotoxic substances, including pro-inflammatory cytokines, reactive oxygen species, and nitric oxide and sustained reactivity of microglia is implicated in the pathology of many neurodegenerative disorders [40]. Inhibition of this process could then protect against neurodegeneration and expansion of brain injury. This view is further supported by epidemiologic data showing that long-term treatment with non-steroidal anti-inflammatory drugs may protect against Alzheimer disease and Parkinson disease [41], [42], [43]. Administration of the bacterial endotoxin LPS in rats induces a consistent glial activation and a subsequent dopaminergic cell loss that parallels many aspects of PD [29], [44], [45]. Here, we show that S14 has potent anti-inflammatory effects *in vivo* after LPS injection in the SNpc. Our results indicate that this compound significantly reduces the accumulation of reactive microglia in the striatum of lesioned-rats. The underlying mechanism of this anti-inflammatory effect of S14 may involve the suppression of certain cytokines, e.g. TNF-α. Indeed our *in vitro* results show that treatment of primary mesencephalic cultures with S14 significantly decreased TNF-α and COX-2 levels, two potent pro-inflammatory agents.

Besides this potent anti-inflammatory action of S14, the administration of this compound also causes a significant preservation of dopaminergic cells loss in the SNpc. PD is characterized by selective degeneration of dopaminergic neurons in the SN. Rats receiving LPS presented classic reductions in the number of TH-immunoreactive cells, a marker of dopaminergic cells in the SN. These animals also demonstrated motor function deficits. A unilateral lesion in nigrostriatal dopaminergic pathway produces an imbalance of dopamine between the lesioned and unlesioned striatum leading to circle toward the side of the lesion [46], [47]. Treatment of the animals with a dopamine agonist such as apomorphine leads to contralateral rotational behavior in denervated animals [48]. This rotational behavior is consistent with damage to dopaminergic neurons in the SN and the decrease of dopamine in the striatum [49]. Behavioral assessment detected significant differences between the LPS and control animals at 3 weeks after LPS administration. S14 administration provided complete protection, as assessed by TH-positive cell number and motor behavioral. We found that apomorphine-induced turning behavior in the LPS-treated group was significantly inhibited by S14 treatment. Overall, the LPS rats treated with S14 were indistinguishable from controls.

In conclusion, here we have shown that inhibition of PDE7 hinders dopaminergic cell death and glial activation in an animal model of PD. The mechanisms that underlie these effects appear to be an elevation of intracellular cAMP, which acts via the PKA-CREB pathway. These results show for the first time that inhibition of the PDE7 enzyme leads to dopaminergic neuronal protection and therefore its inhibitors may exert useful therapeutic actions in patients with PD, a hypothesis that is amenable to clinical testing.

## Materials and Methods

### Animal experiments

All procedures with animals were specifically approved by the ‘Ethics Committee for Animal Experimentation’ of the Instituto de Investigaciones Biomedicas (CSIC-UAM), licence number SAF 2010/16365, and carried out in accordance with the protocols issued which followed National (normative 1201/2005) and International recommendations (normative 86/609 from the European Communities Council). Adequate measures were taken to minimize pain or discomfort of animals.

### LPS injection *in vivo*

Adult male Wistar rats (8–12 weeks old) were used in this study. The animals, divided into four groups, with at least six rats in each group, were properly anaesthetized and placed in a stereotaxic apparatus (Kopf Instruments, CA). LPS (10 µg in 2.5 µl PBS) alone or in combination with S14 (20 nmol) or with BRL50481 (60 nmol) were injected into the right side of the SNpc (coordinates from Bregma: posterior - 4.8 mm; lateral + 2.0 mm; ventral: +8.2 mm, according to the atlas of Paxinos and Watson [50]). The dose of LPS was chosen based in previous published data [29], [30], [51]. The amount of S14 injected was calculated taking into account the distribution volume of this cerebral area and the effective dose observed in the *in vitro* experiments. Control animals of the same age were injected with PBS. Rats were then housed individually to recover and sacrificed 72 h after lesioning.

### Histology and Immunohistochemistry

Seventy-two hours after lesioning, the animals were anaesthetized and perfused transcardially with a 4% paraformaldehyde solution. The brains were removed, postfixed in the same solution at 4°C overnight, cryoprotected, frozen, and 30 µm coronal sections were obtained in a cryostat. Free-floating sections were processed for cresyl violet (Nissl stain) or immunohistochemistry using the diaminobenzidine method as previously described [52]. To detect PDE7 in SNpc, rabbit anti-PDE7A and goat anti-PDE7B antibodies (Santa Cruz Biotech) were used. For immunodetection of activated glia and dopaminergic neurons, a mouse anti-CD11b antibody (Serotec, Germany) and a rabbit anti-tyrosine hydroxylase (Chemicon/Millipore, USA) antibody, respectively, were used. After being dehydrated, cleared, and mounted with DePeX (Serva, Heidelberg, Germany), samples were examined with a Zeiss (Oberkochen, Germany) Axiophot microscope, equipped with an Olympus DP-50 digital camera, and a Leica (Nussloch, Germany) MZ6 modular stereomicroscope. Four animals from each experimental group were analyzed. Neuronal integrity and specifically dopaminergic cell death was assessed by counting the percentage of Nissl-stained and TH^+^ cells, respectively, in the SNpc in four well-defined high magnification (x400) fields per animal, using a computer-assisted image analySIS software (Soft Imaging System Corp). Microgliosis was quantified similarly.

#### Behavioural testing

Apomorphine-induced rotational behavioural test was performed 3 weeks following LPS lesioning of the SNpc. Rats were given a subcutaneous injection of apomorphine (0.5 mg/kg in saline), then placed individually in plastic beakers and videotaped for 30 minutes. Analysis of completed (360°) rotations was made offline and expressed as number of turns per minute. Rats showing more than six turns per minute were considered as properly lesioned. Three different experiments with al least 12 animals/experimental group were performed.

### Mesencephalic cell cultures

Cultures were derived from the ventral mesencephalon of rat embryos at embryonic day 14. Briefly, rats were killed by cervical dislocation and embryonic sacs dissected and collected in ice-cold HBSS medium (Ca^2+^ and Mg^2+^ free). Ventral mesencephalon was isolated, gently minced and triturated with a micropipette in HBSS medium. Then the supernatant was collected, and centrifuged at 1200 x*g*/5 min. The pellet was resuspended in culture media (MEM supplemented with 10% FBS, 10% HS, glucose 1 g/l glutamine 2 mM, sodium pyruvate 1 mM, non-essential aminoacids 100 µM, penicillin 50 U/ml and streptomycin 50 µg/ml), and cells seeded onto 24-well plates (5×10^5^ cells/well) or 96-well plates (1×10^5^ cells/well). After 1 week in culture, cells were treated with LPS (1 µg/ml) [53], [54], [55] or 6-OHDA (35 µM, Sigma), alone or in combination with S14 (10 µM). The effective dose of S14 was determined based on previous studies on EC50 [22]. After 24 h, cultures were processed for immunocytochemistry and nitrite determination.

### SH-SY5Y cell culture

The human neuroblastoma SH-SY5Y cell line was obtained from Sigma-Aldrich and propagated in F12 medium/EMEM containing glutamine (2 mM), 1% of non-essential amino acids and 15% of fetal bovine serum (FBS), under humidified 5% CO_2_ and 95% air. On attaining semiconfluence, cells were treated or not with 6-OHDA (35 µM, Sigma) for 24 h. Some cultures were pretreated for 1 h with S14 (10 µM), Rolipram (30 µM, Tocris Bioscience) or BRL-50481 (30 µM, Tocris Bioscience). To analyze the role of cAMP, some plates were also preincubated with the PKA inhibitor H-89 (20 µM, BIOMOL Research Laboratories) or the cAMP antagonist Rp-cAMP (100 µM, BIOMOL Research Laboratories) for 24 h before the addition of the different compounds. At different times after treatments, cells were processed for western blot, cell viability assay, LDH measurement, nitrites release, and immunocytochemical analysis.

### Cell viability assay

Cell viability was measured using the MTT assay (Roche Diagnostic, GmbH), based on the ability of viable cells to reduce yellow MTT to blue formazan. Briefly, cells were cultured in 96-well plates and treated with the indicated compounds for 16 h, then cells were incubated with MTT (0.5 mg/ml, 4 h) and subsequently solubilized in 10% SDS/0.01 M HCl for 12 h in the dark. The extent of reduction of MTT was quantified by absorbance measurement at 595 nm according to the manufacturer's protocol.

### LDH release assay

Cytotoxicity was assessed by measuring the levels of lactate dehydrogenase (LDH) released into the culture medium 16 h after the different treatments. LDH activity was measured using a Cytotoxicity Detection kit (Roche Molecular Biochemicals, Indianapolis, IN, USA) and quantified by measuring absorbance at 490 nm.

### Nitrites measurement

Accumulation of nitrites in media was assayed by the standard Griess reaction. After stimulation of cells with the different treatments for 16 hours, supernatants were collected and mixed with an equal volume of Griess reagent (Sigma- Aldrich). Samples were then incubated at room temperature for 15 minutes and absorbance read using a plate reader at 492/540 nm.

### cAMP assay

Quantification of cAMP was carried out using the EIA (enzyme immunoassay) kit from GE Healthcare. Briefly, SH-SY5Y cells were seeded at 3×10^4^/well in 96-well dishes and incubated overnight before the assay. After 1 h incubation with S14, Rolipram or BRL50481, cAMP intracellular levels were determined following the manufacture's instructions.

### Immunoblot analysis

Proteins were isolated from brain tissue or cell cultures by standard methods. Some SH-SY5Y cultures were pre-treated with Rolipram (30 µM), BRL50481 (BRL, 30 µM), or S14 (10 µM) for 1 h before 6-OHDA (35 µM) addition and kept in these conditions for 16 h. A total amount of 30 µg of protein was loaded on a 10% SDS-PAGE gel. After electrophoresis, proteins were transferred to nitrocellulose membranes (Protran, Whatman, Dassel, Germany) and blots were probed with the indicated primary antibodies, as previously described [56]. The antibodies used were the following: rabbit anti-PDE7A (1∶1000, Santa Cruz Biotech., USA), goat anti-PDE7B (1∶1000; Santa Cruz Biotech., USA), mouse monoclonal anti-α-tubulin (1∶5000; Sigma), rabbit anti-p-CREB (1∶1000; Cell Signaling) and rabbit anti-CREB (1∶1000; Cell Signaling). All incubations with primary antibodies were carried out overnight, with gently shaking at 4°C. Secondary peroxidase-conjugated donkey anti-rabbit and rabbit anti-mouse antibodies were from Amersham Biosciences (GE Healthcare, Buckinghamshire, England) and Jackson Immunoresearch, respectively. Secondary antibodies incubations were done at room temperature for 1 hour. Values in figures are the average of the quantification of at least three independent experiments corresponding to three different samples.

### Immunocytochemistry

At the end of the treatment period, SH-SY5Y or primary mesencephalic cultures, grown on glass cover-slips in 24-well cell culture plates, were washed with PBS and fixed for 30 min with 4% paraformaldehyde at 25°C and permeabilized with 0.1% Triton X-100 for 30 min at 37°C. After 1 h incubation with the corresponding primary antibody, cells were washed with PBS and incubated with an Alexa-labeled secondary antibody (Invitrogen, San Diego, CA) for 45 min at 37°C. Images were acquired using a Radiance 2100 confocal microscope (Bio-Rad, Hercules, CA), with a 350 nm diode laser to excite DAPI (4,6,diamidino-2-phenylindole) a 488-Argon laser to excite Alexa 488 and a 647 laser to excite Alexa 647. Confocal microscope settings were adjusted to produce the optimum signal-to-noise ratio. To compare fluorescence signals from different preparations, settings were fixed for all samples within the same analysis. The following antibodies were used: rabbit anti-PDE7A (Santa Cruz Biotech), goat anti-PDE7B (Santa Cruz Biotech), goat anti-Cox-2 (Santa Cruz Biotech), goat anti-TNFα (Santa Cruz Biotech), rabbit anti-tyrosine hydroxylase (Chemicon/Millipore), mouse anti-tyrosine hydroxilase (Sigma) and mouse anti-NeuN (Chemicon). For the quantification of COX-2, TNF-α and TH immunoreactive cells, the number of positive cells was quantified in 20 independent random fields at x400 magnification.

#### Measurement of apoptosis

To calculate the extend of apoptotic cell death, SH-SY5Y cells were treated or not with S14, incubated with 6-OHDA for 16 h, and phosphatydilserine (PS) exposure on the surface of apoptotic cells was detected by confocal microscopy after staining with Annexin V-FITC (Bender MedSystems, Vienna, Austria). Levels of active caspase-3 were also determined using a specific rabbit anti-active caspase-3 antibody (R&D Systems). For the quantification of Annexin-V-positive cells and active caspase-3 immunoreactive cells, the number of positive cells was quantified as described above.

### Statistics analysis

Statistical comparisons for significance among differents groups of animals were performed by ANOVA followed by Newman-Keuls'test for multiple comparisons. Student's *t*-test was used to analyze statistical differences between cells. Differences were considered statistically significant at *p*<0.05.
